# Venous Thrombogenesis and Cervical Cancer: Plasma MicroRNAs as Prognostic Indicators of Tumor Behavior

**DOI:** 10.3390/ijms26199796

**Published:** 2025-10-08

**Authors:** Mariana Teixeira Costa, Beatriz Vieira Neto, José Brito da Silva, Luísa Carvalho, Lurdes Salgado, Deolinda Pereira, Filomena Adega, Valéria Tavares, Rui Medeiros

**Affiliations:** 1 Molecular Oncology and Viral Pathology Group, Research Center of IPO Porto (CI-IPOP)/CI-IPOP@RISE (Health Research Network), Portuguese Oncology Institute of Porto (IPO Porto)/Pathology and Laboratory Medicine Department/Clinical Pathology/Porto Comprehensive Cancer Center Raquel Seruca (Porto.CCC), 4200-072 Porto, Portugal; mariana.t.costa@ipoporto.min-saude.pt (M.T.C.); beapedro7@hotmail.com (B.V.N.); valeria.tavares@ipoporto.min-saude.pt (V.T.); 2 CytoGenomics Lab, Department of Genetics and Biotechnology, University of Trás-os-Montes and Alto Douro, 5000-801 Vila Real, Portugal; filadega@utad.pt; 3 Research Department, Portuguese League Against Cancer (NRNorte), 4200-172 Porto, Portugal; 4 Oncology Department, Portuguese Institute of Oncology of Porto (IPO Porto), 4200-072 Porto, Portugal; jose.brito.silva@ipoporto.min-saude.pt (J.B.d.S.); dpereira@ipoporto.min-saude.pt (D.P.); 5 External Radiotherapy Department, Portuguese Institute of Oncology of Porto (IPO Porto), 4200-072 Porto, Portugal; luisa.pinto@ipoporto.min-saude.pt (L.C.); lurdes.salgado@gmail.com (L.S.); 6 Biosystems and Integrative Sciences Institute (BioISI), Faculty of Sciences, University of Lisbon, 1749-016 Lisboa, Portugal; 7 Abel Salazar Institute for the Biomedical Sciences (ICBAS), University of Porto, 4050-313 Porto, Portugal; 8 Biomedical Research Center (CEBIMED), Faculty of Health Sciences, Fernando Pessoa University, 4200-150 Porto, Portugal

**Keywords:** cervical cancer, venous thromboembolism, cancer-associated thrombosis, microRNAs, biomarkers, prognosis

## Abstract

Cervical cancer (CC) is the fourth most common cancer among women globally, with venous thromboembolism (VTE) representing a life-threatening complication. Cancer-associated thrombosis (CAT) arises from tumor-driven activation of hemostasis, worsening prognosis. Recently, circulating microRNAs (miRNAs) have emerged as potential biomarkers for both CAT and cervical tumorigenesis. Thus, this study aimed to assess the implications of five miRNAs—miR-20a-5p, -23a-3p, -125b-5p, -145-5p, and -616-3p—in CC-related VTE context. These miRNAs were quantified by RT-qPCR in plasma from 69 CC patients before treatment. Briefly, VTE occurred in nine patients, decreasing overall survival (OS) [log-rank test, *p* = 0.005; hazard ratio (HR) = 4.78; 95% confidence interval (CI), 1.42–16.05]. Lower miR-20a-5p levels predicted VTE (ꭓ^2^ test, *p* = 0.027) and, in subgroup analyses, they were linked to cervical squamous cell carcinoma (CSCC) and older age (ꭓ^2^ test, *p* = 0.003 and *p* = 0.043, respectively). In VTE patients, miR-145-5p downregulation was associated with improved OS (log-rank test, *p* = 0.018), an effect also observed in the adenocarcinoma (ADC) subgroup (log-rank test, *p* = 0.039). The remaining miRNAs showed subtype-specific links to clinicopathological features and survival. These findings highlight the potential value of circulating miRNAs in thrombotic risk and prognosis assessment in CC.

## 1. Introduction

Cervical cancer (CC) ranks as the fourth most diagnosed cancer and the fourth leading cause of cancer-related death among women worldwide, with approximately 660,000 new cases and 350,000 deaths in 2022 [1,2]. The disease mainly results from persistent infection with high-risk human papillomavirus (HPV), particularly types 16 and 18 [3,4]. Histologically, CC includes two main subtypes: squamous cell carcinoma (CSCC), which is the most common but with a decreasing incidence due to effective prevention through HPV vaccination and early detection, and adenocarcinoma (ADC) [5,6,7].

Despite the decrease in CC incidence and mortality, especially in high-income countries with organized vaccination and screening programs [8,9], CC patients face additional challenges, notably cancer-associated thrombosis (CAT) [10]. This condition mainly involves venous thromboembolism (VTE) events, which occur at a significantly higher rate among cancer patients (up to seven-fold compared to the general population), and it is recognized as the second leading cause of death in this subpopulation [11,12,13,14]. In CC, the average CAT incidence ranges between 9% and 12% and is linked to poor survival [15,16]. This increased pro-thrombotic risk reflects the ability of CC cells to release pro-coagulant, pro-angiogenic and inflammatory factors that enhance tumor fitness and facilitate disease progression. Combined with the effects of tumor treatments, these changes sustain a pro-thrombotic state, ultimately contributing to the clinical manifestation of CAT [17,18,19].

Regarding the disease pathogenesis, thrombosis is defined as the formation of an abnormal blood clot (thrombus) within a blood vessel, which can partially or completely block blood flow [20]. Under normal physiological conditions, hemostasis maintains vascular integrity after injury, allowing normal blood flow through vasoconstriction, primary hemostasis (platelet adhesion and aggregation) and secondary hemostasis (activation of the coagulation cascade to form a stable fibrin clot) [21]. The coagulation cascade proceeds through the extrinsic pathway, triggered by tissue factor (TF) exposure, and the intrinsic pathway, activated by contact with negatively charged surfaces [22,23,24]. Both converge on thrombin generation, which converts fibrinogen into fibrin, stabilizing the platelet plug—common coagulation cascade [25,26]. Once vascular repair begins, fibrinolysis removes the clot through plasmin-mediated degradation of fibrin [27]. Several inhibitory mechanisms operate to prevent excessive coagulation [28]. One of them is tissue factor pathway inhibitor 1 (TFPI1 or TFPI), which binds and inhibits the TF-activated coagulation factor VII (FVIIa) complex in an activated coagulation factor X (FXa)-dependent manner, thereby controlling initiation of the extrinsic coagulation pathway and limiting excessive thrombin and fibrin generation [29]. Its homolog, tissue factor pathway inhibitor 2 (TFPI2), is involved in the regulation of intrinsic clotting by targeting plasma kallikrein (pKLK) and activated coagulation factor XI (FXIa). However, it also modulates fibrinolysis by targeting plasmin [29,30,31].

MicroRNAs (miRNAs) are small non-coding RNAs (ncRNAs) that regulate gene expression post-transcriptionally, influencing multiple biological pathways, including hemostasis [32]. A defining feature of miRNA activity is that each miRNA can regulate the expression of multiple messenger RNAs (mRNAs), and, conversely, a single transcript can be regulated by different miRNAs [33,34,35]. Beyond their intracellular roles, these ncRNAs can be found circulating in body fluids, including plasma and serum. Their extracellular presence is largely attributed to their remarkable stability and resistance to degradation by endogenous RNases, making them valuable biomarkers [36,37]. Focusing on hemostasis, miRNAs are key regulators of the coagulation system by directly targeting hemostatic genes such as *fibrinogen* (*coagulation factor I*), *coagulation factor 11* (*F11*) and *coagulation factor 3* (*F3)* (encoding TF protein), as well as anticoagulants such as *TFPI*, *antithrombin* and *plasminogen activator inhibitor-1* (*PAI-1*) [36,38,39].

Given the central role of the TF pathway in CAT onset, miRNAs targeting *F3*, *TFPI* and *TFPI2* may provide prognostic value among CC patients. Previously, our research group conducted a comprehensive review of these miRNAs [29]. More recently, an exhaustive review of the existing literature on miRNAs involved in cervical carcinogenesis was conducted. Considering both assessments, miR-20a-5p, -23a-3p, -125b-5p, -145-5p and -616-3p emerge as potential multipurpose biomarkers for early CC diagnosis, prognostication, CAT risk stratification and therapeutic guidance in CC patients. The present study aimed to evaluate the implications of these five miRNAs in a cohort of 69 CC patients from the Northern Region of Portugal, considering the bidirectional interplay between tumor progression and thrombosis. More specifically, the impact of these circulating miRNAs on the development of venous thrombotic events and patient prognosis, independently of VTE, was examined. To our knowledge, this is the first study to explore the potential role of miRNAs in linking TF pathway to both CAT occurrence and CC behavior, highlighting their prognostic relevance in CC patients.

## 2. Results

### 2.1. Correlation Between Baseline miRNA Expression

**Figure 1:**
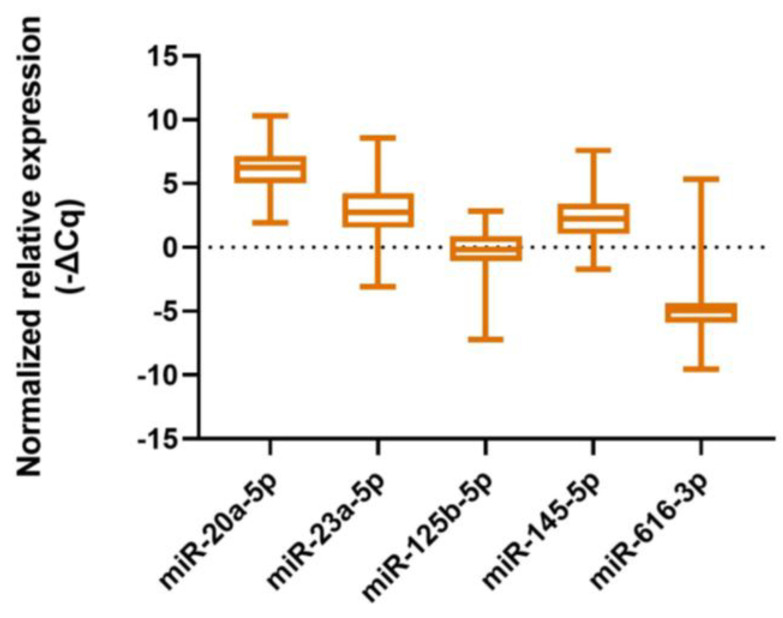
Normalized relative expression of evaluated miRNAs represented as -ΔCq values.

Except for miR-616-3p, which only showed moderate positive correlations with miR-20a-5p (ρ = 0.555), all pairwise correlations between plasma miRNA expression levels were significant and positive, with coefficients ≥ 0.500 (Figure 1 and Table 1). The strongest correlation was observed among miR-23a-3p and miR-145-5p (Pearson’s r = 0.700).

### 2.2. MiRNA Expression Levels and Patients’ Characteristics

The distribution of miRNA expression varied significantly across patient characteristics, except for miR-616-3p (Table 2). Reduced miR-20a-5p levels were more common in older patients (≥52 years) and those with CSCC tumors (ꭓ^2^ test, *p* = 0.043 and *p* = 0.003, respectively). Patients at early International Federation of Gynecology and Obstetrics (FIGO) stages (<IIB stages) more commonly exhibited low-to-medium expression levels of miR-23a-3p (ꭓ^2^ test, *p* = 0.025). Regarding miR-125b-5p, patients with ADC tumors tended to display higher miRNA expression levels (ꭓ^2^ test, *p* = 0.018). As for miR-145-5p, its lower expression was more common among patients with CSCC and those at early disease stages (ꭓ^2^ test, *p* = 0.027 and *p* = 0.011, respectively).

### 2.3. MiRNA Expression Levels and CC-Related VTE Incidence

**Figure 2:**
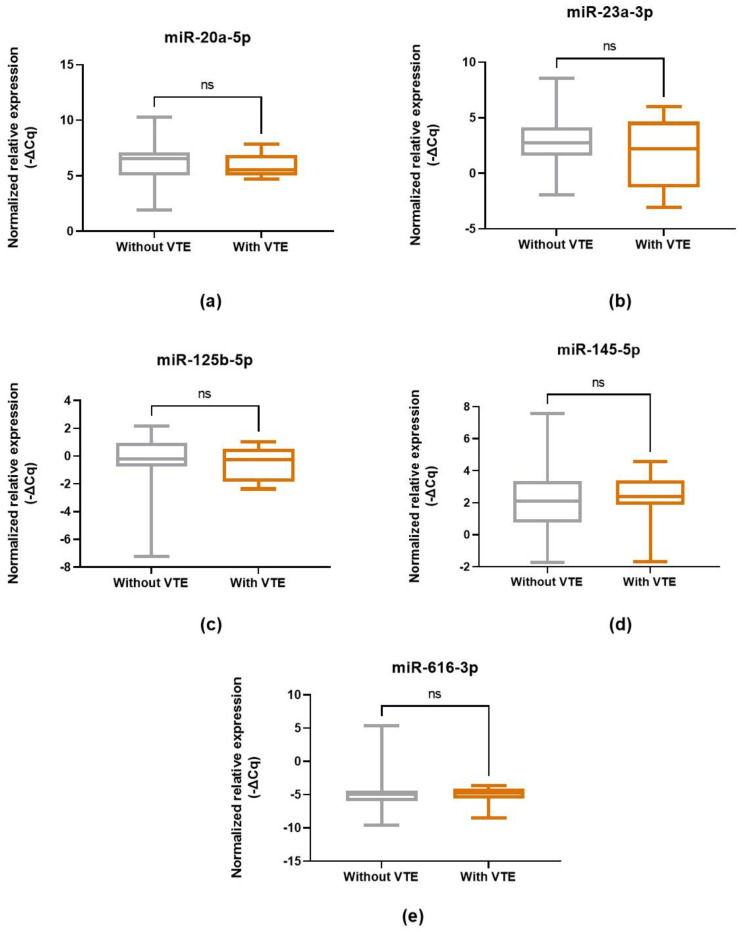
Normalized miRNA expression levels (-ΔCq) in CC patients prior to first-line chemotherapy, based on VTE status. Unpaired *t*-test: (**a**) miR-20a-5p, (**b**) miR-23a-3p, (**c**) miR-125b-5p, (**d**) miR-145-5p. Mann–Whitney U test: (**e**) miR-616-3p. Ns, non-significant.

The association of the evaluated miRNAs with VTE status in CC patients [without VTE versus (vs.) with VTE after CC diagnosis] was initially assessed using the Mann–Whitney U test or an unpaired *t*-test, depending on data normality. There were no associations between the miRNAs and VTE development (Figure 2). However, as a subsequent analysis, a ꭓ^2^ test was performed to explore whether the expression pattern of the miRNAs vary according to VTE status. This analysis revealed that VTE-free patients more frequently presented an elevated expression of miR-20a-5p (expression profile 1—see Section 4.7. Statistical Analysis) compared to those who developed the condition after CC diagnosis (72.0% and 22.2%, respectively; ꭓ^2^ test, *p* = 0.012). No further significant differences were observed for the remaining miRNAs (ꭓ^2^ test, *p* > 0.05). Consistently, logistic regression analysis corroborated the association between miR-20a-5p and VTE status. Specifically, those with high levels of this miRNA (expression profile 1) exhibited a reduced likelihood of VTE [odds ratio (OR) = 0.12; 95% confidence interval (CI), 0.02–0.71], indicating a potential protective role of this miRNA. No further significant associations were detected (*p* > 0.05).

### 2.4. VTE Status and Patients’ Prognosis

**Figure 3:**
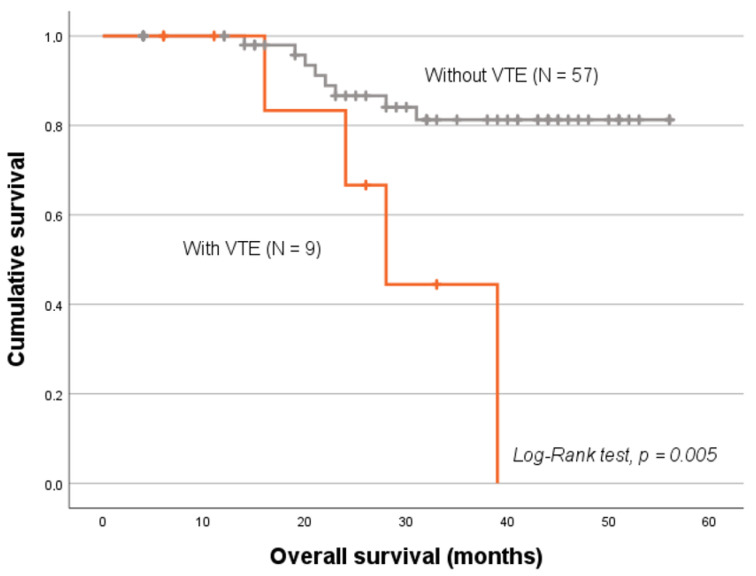
Kaplan–Meier analysis of 10-year OS in CC patients based on VTE status. Patients with the condition had a decreased OS compared to those without (mean OS of 30.2 ± 4.3 months and 49.8 ± 2.0 months, respectively, log-rank test, *p* = 0.005).

The occurrence of venous thrombotic events among CC patients was shown to negatively impact their survival (log-rank test, *p* = 0.005; Figure 3). Considering only the patients with available miRNA expression data (N = 66), those who developed the condition after cancer diagnosis (N = 9) had a markedly shorter mean overall survival (OS) (30.2 ± 4.3 months) compared to their counterparts (49.8 ± 2.0 months). Indeed, developing VTE after CC diagnosis was linked to a nearly fivefold increased risk of death within 10 years [hazard ratio (HR) = 4.78; 95% CI, 1.42–16.05; *p* = 0.011].

### 2.5. MiRNA Expression Levels and Patients’ Prognosis

**Figure 4:**
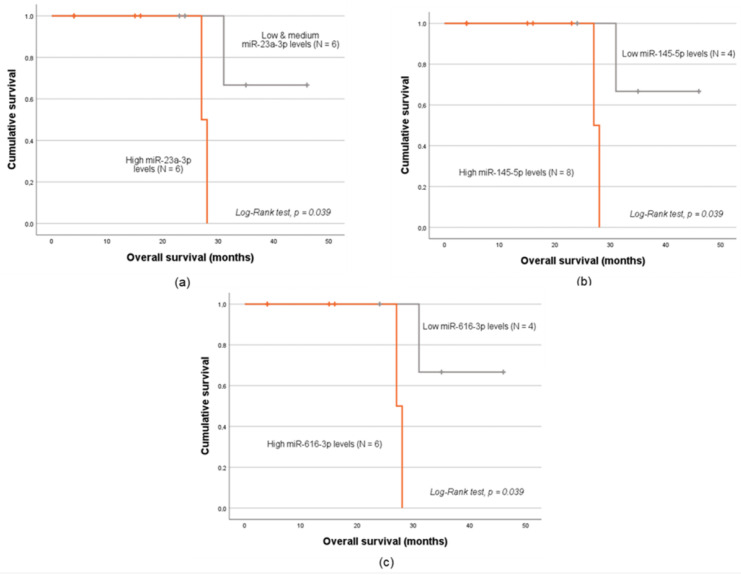
Kaplan–Meier analysis of 10-year OS of ADC patients. Those with low levels of miR-23a-3p (**a**), low levels of miR-145 (**b**) and low levels of miR-616-3p (**c**) had a higher OS than their respective counterparts (mean OS of 41.0 ± 4.1 months and 27.5 ± 0.5 months, respectively, log-rank test, *p* = 0.039).

Considering the entire cohort (N = 66), no significant association was exhibited between baseline expression levels of any of the miRNAs studied and patients’ OS. In contrast, significant associations were identified in the subgroup analyses. Namely, among patients with ADC tumors, a higher OS was observed for those with reduced levels of miR-23a-3p, miR-145-5p and miR-616-3p (log-rank test, *p* < 0.05; Figure 4a, Figure 4b and Figure 4c, respectively).

**Figure 5:**
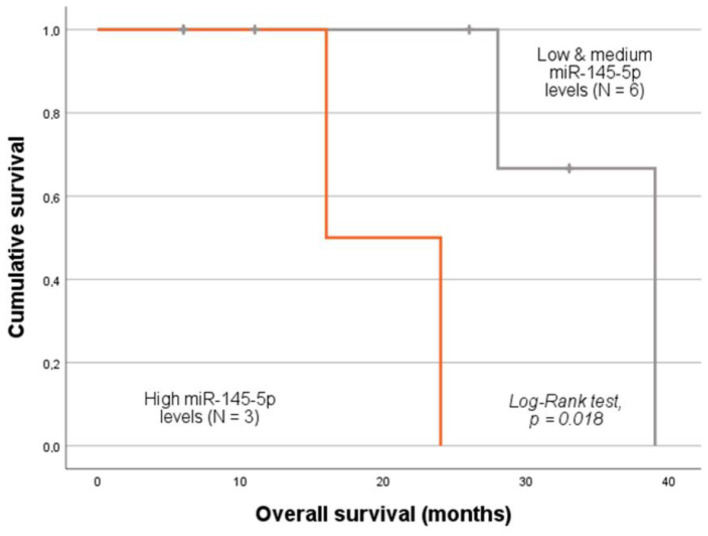
Kaplan–Meier analysis of 10-year OS of CC patients with clinical evidence of VTE. Patients with low levels of miR-145-5p had a higher OS than their counterparts (mean OS of 35.3 ± 4.2 months and 20.0 ± 4.0 months, respectively, log-rank test, *p* = 0.018).

In the stratified analysis according to VTE status, in the subgroup of patients with clinical evidence of thrombosis occurrence, those with low-to-medium plasma levels of miR-145-5p had a higher OS (log-rank test, *p* = 0.018; Figure 5). As for the subgroup analyses considering patients’ age and FIGO stage, no statistically significant association between miRNA baseline expression levels and patient OS was observed (log-rank test or Tarone–Ware test, *p* > 0.05).

### 2.6. In Silico Analysis

**Figure 6:**
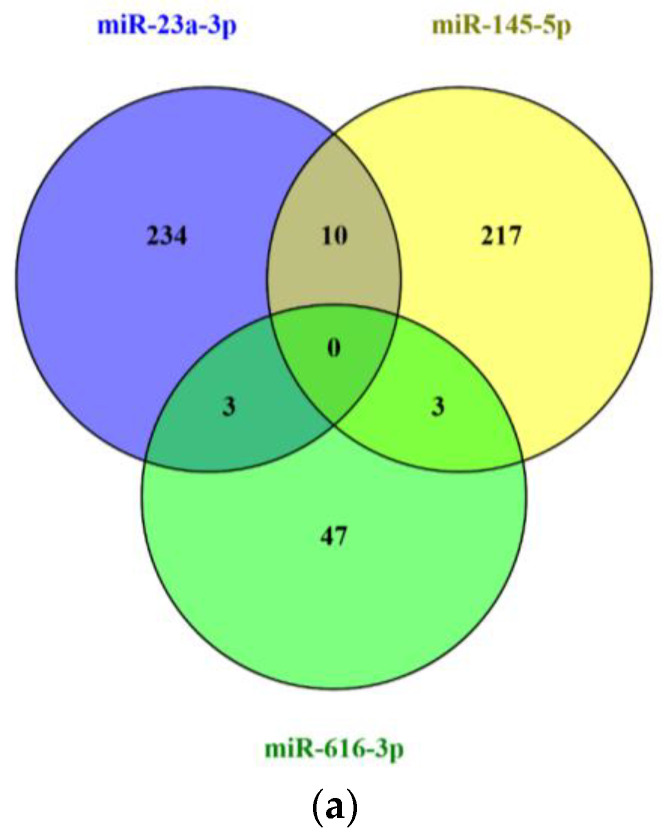

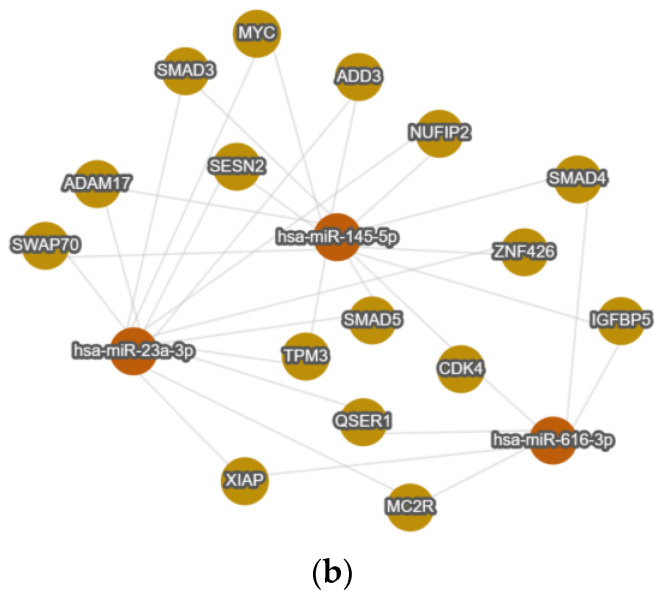
Shared targets of hsa-miR-23a-3p, hsa-miR-145-5p, and hsa-miR-616-3p. Venn diagram of the validated targets of each miRNA constructed using Venny 2.1 (https://bioinfogp.cnb.csic.es/tools/venny/; last accessed on 9 August 2025) (**a**). Representation of the shared targets using miRTargetLink 2.0 database (https://ccb-compute.cs.uni-saarland.de/mirtargetlink2/; last accessed on 9 August 2025) (**b**).

**Figure 7:**
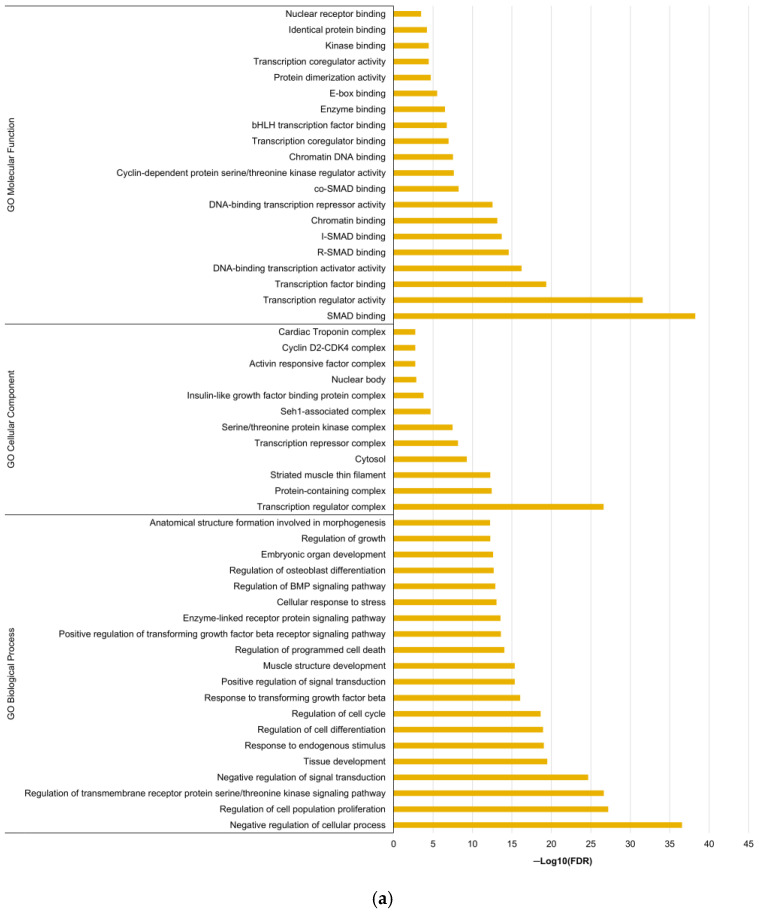

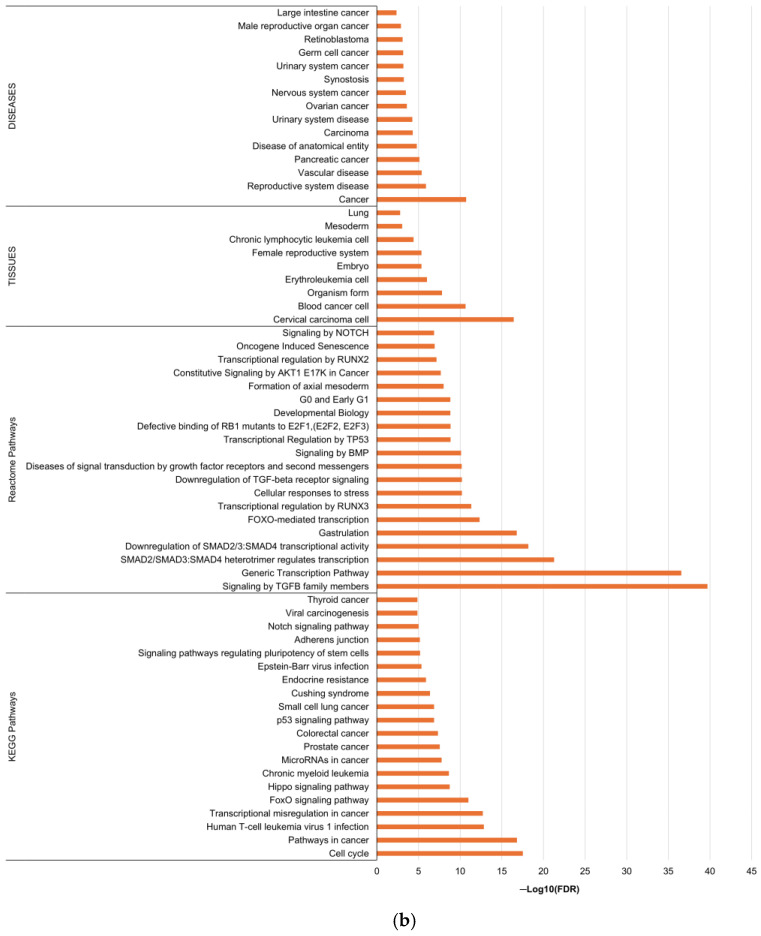
Functional enrichment analysis. The top 20 enriched terms for GO (**a**), DISEASES, TISSUES, KEGG pathways, and Reactome pathways (**b**) associated with the shared targets of hsa-miR-23a-3p, hsa-miR-145-5p, and hsa-miR-616-3p (FDR < 0.01). Targets were identified using miRTargetLink 2.0, and the PPI network was generated via STRINGapp in Cytoscape 3.10.3.

Given the impact of miR-23a-3p, miR-145-5p, and miR-616-3p on patient prognosis, the shared biological implications of these miRNAs were explored through an in silico analysis. First, validated targets of each miRNA (247, 230 and 53 targets for miR-23a-3p, miR-145-5p, and miR-616-3p, respectively) were identified using miRTargetLink 2.0 (Appendix A Table A1). Focusing on the targets shared by at least two miRNAs (Figure 6), the STRINGapp Protein Query from Cytoscape 3.10.3 was employed to generate a physical subnetwork of protein–protein interaction (PPI). The top 20 enriched terms for Gene Ontology (GO) categories, DISEASES, TISSUES, KEGG pathways, and Reactome pathways were retrieved after conducting a functional enrichment analysis (Figure 7).

## 3. Discussion

Although the global incidence of CC has declined, it remains a major cause of cancer-related death in women, particularly when diagnosed at advanced stages, where treatment options are limited [2]. A personalized approach to CC management requires a comprehensive understanding of the tumor microenvironment, including how the disease interacts with the hemostatic system. VTE is a common complication in cancer, worsening prognosis, and in CC, it is associated with particularly poor survival [43,44,45]. This underscores the need for CC biomarkers to guide clinical decisions, while aiding VTE risk prediction [46]. In this regard, miRNAs recently emerged as promising candidates. Hence, this study investigated circulating miRNAs in CC patients undergoing concurrent chemoradiotherapy (CCRT), aiming to identify ncRNAs associated with both CAT and tumor aggressiveness to support personalized treatment strategies.

In this study, 13% of CC patients developed clinically evident VTE, though the absence of active screening suggests that the true incidence may be higher. This aligns with previous reports showing thromboembolic rates of 9-12% in CC patients, confirming their elevated thrombotic tendency [15,16]. Importantly, VTE was associated with significantly poorer survival, with affected patients showing a shorter mean OS than those without thrombotic complications (30.2 ± 4.3 months and 49.8 ± 2.0 months, respectively; log-rank test, *p* = 0.005), Consistently, VTE patients exhibited nearly a five-fold higher risk of death within 10 years (95% CI, 1.42–16.05; *p* = 0.011).

Among the miRNAs analyzed—miR-20a-5p, -23a-3p, -125b-5p, -145-5p and -616-3p—only miR-23a-3p and miR-145-5p showed a strong positive correlation (Pearson’s *r* = 0.700; *p* < 0.001), likely reflecting overlapping regulatory roles. Consistently, the in silico analysis further revealed that these miRNAs share multiple predicted target genes, which reinforces the likelihood of coordinated regulation in vivo. Consistently, both miRNAs have been previously implicated in the regulation of hemostasis and CC progression. In the general population with VTE, downregulation of miR-145-5p has been found to increase *F3* expression, thus increasing TF levels, which promotes thrombus formation [47,48]. In turn, miR-23a is reported to target *TFPI2*, which encodes a regulatory protein involved not only in thrombin formation (inhibiting TF coagulation pathway) but also in platelet activation and fibrinolysis [49,50]. Parallelly, TFPI2 has been associated with CAT, where its high levels impair fibrinolysis, promoting the maintenance and progression of established thrombus. This was observed in epithelial ovarian cancer patients with asymptomatic VTE and a positive D-dimer result [31]. In CC, TFPI2 acts as a modulator of tumor progression. Namely, its downregulation through miR-23a is reported to contribute to angiogenesis in CC cells [51,52,53,54]. Similarly, miR-145-5p functions as a tumor suppressor by regulating oncogenic pathways [55]. Overall, the positive correlation observed between miR-23a-3p and miR-145-5p (Pearson’s *r* = 0.700; *p* < 0.001) likely reflects coordinated regulation of hemostasis within the tumor microenvironment, as they modulate the expression of complementary components of the coagulation pathway (TFPI2 and TF, respectively), with potential implications for both VTE risk and tumor progression.

Several moderate correlations were observed among the miRNAs, particularly between miR-20a-5p and miR-23a-3p (Pearson’s *r* = 0.659; *p* < 0.001), miR-20a-5p and miR-145-5p (Pearson’s *r* = 0.662; *p* < 0.001), and miR-125b-5p and miR-145-5p (Pearson’s *r* = 0.660; *p* < 0.001). These correlations are biologically plausible, since miR-20a-5p, miR-23a-3p and miR-145-5p all participate in the regulation of the TF pathway. Namely, miR-145-5p directly targets *F3* [48], miR-23a-3p inhibits *TFPI2* [49,56] and miR-20a-5p has been validated as another *F3* regulator [57,58], with a previous in silico analysis suggesting that it may additionally target *TFPI2* [56]. The shared roles of these miRNAs in TF-related processes likely underline their correlated expression. Regarding the correlation between miR-125b-5p and miR-145-5p, it may reflect their contribution to a pro-thrombotic microenvironment. Notably, miR-125b influences the expression of coagulation factor IX (encoded by the *F9* gene) [59,60]. Both miR-125b-5p and miR-145-5p show decreased expression during HPV-driven cervical lesion progression, suggesting common regulation by viral infection [61,62,63]. However, their involvement in overlapping molecular pathways relevant to cervical carcinogenesis is poorly explored. As for miR-616-3p, it displayed no significant correlations, except with miR-20a-5p, possibly due to their shared *TFPI2* modulation [47,56].

Considering both the entire cohort and subgroup analysis, miR-20a-5p and miR-145-5p emerged as the most relevant miRNAs in this study. Regarding miR-20a-5p, its low expression levels were significantly associated with VTE development in CC patients (OR = 0.12; 95% CI, 0.02–0.71), suggesting a role in CAT, consistent with its known function in suppressing TF levels [57,58]. Beyond coagulation, miR-20a-5p, a member of the oncogenic miR-17-92 cluster, has been linked to invasion and metastasis [64]. Previous reports indicate its higher expression in CC compared to healthy controls [65]. In this study, while no direct impact of miR-20a-5p expression pattern on CC patients’ OS was observed, the miRNA expression significantly varied depending on important clinicopathological features. Namely, reduced miR-20a-5p levels were more common in older patients (≥52 years) and those with CSCC tumors (ꭓ^2^ test, *p* = 0.043 and *p* = 0.003, respectively). The first observation suggests an age-related difference in the miRNA regulation, highlighting age at CC diagnosis as a potentially relevant factor influencing miR-20a-5p expression. This is consistent with previous studies showing that the miR-17-92 cluster tends to be downregulated with aging, as its lower levels correlate with senescence-associated pathways [66]. Regarding the link between miR-20a-5p and CSCC tumors, though an underlying biological mechanism cannot be entirely dismissed, this finding was most likely influenced by the predominance of CSCC cases among patients who developed VTE. Notably, although no association between histological subtype and VTE was observed, all VTE cases occurred among CSCC patients.

MiR-145-5p expression pattern significantly contrasted depending on several clinicopathological features and survival outcomes in CC, but its role appears context- and histology-dependent. In this study, downregulation of miR-145-5p was more frequent among patients with CSCC tumors (ꭓ^2^ test, *p* = 0.027). In agreement, a prior study showed reduced expression in both tissue and plasma of CSCC patients [63]. Interestingly, the same report linked lower miR-145-5p levels to advanced FIGO stages, while in this cohort, the miRNA downregulation was more common in patients with early-stage disease (ꭓ^2^ test, *p* = 0.011). This discrepancy may be explained by differences in cohort characteristics. Further studies with larger cohorts are needed to clarify this finding.

Regarding patient survival, miR-145-5p expression showed no significant association with OS considering the entire cohort. However, stratified analysis focusing on ADC patients revealed that lower miRNA levels were associated with better outcomes (log-rank test, *p* = 0.039), indicating potential histology-specific effects. Similar patterns have been reported in esophageal cancer (EC), where miR-145 suppresses tumorigenesis in esophageal squamous carcinoma (ESCC) but promotes invasion and anoikis resistance in esophageal adenocarcinoma (EAC) [67]. This dual role may be explained by its regulation of *c-Myc*, a repressor of integrins. By targeting *c-Myc*, miR-145 indirectly increases integrin expression, which enhances tumor cell adhesion and invasion, ultimately contributing to a poorer prognosis [68]. Given that HPV infection is also a possible cause of EC, similar underlying mechanisms may also be relevant in CC. Additional investigation is needed to explore the histology-dependent expression of miR-145-5p in cervical tumorigenesis. In the VTE subgroup, patients with lower-to-medium miR-145-5p levels exhibited better OS compared to those with high levels (log-rank test, *p* = 0.018). Once again, this seemingly paradoxical finding may reflect histological subtype and disease stage distribution, as patients with lower miR-145-5p were more likely to have CSCC and earlier-stage tumors, both generally associated with better prognosis [7]. However, the prognostic impact of histology remains controversial. While several studies suggest that CSCC is associated with better prognosis compared to ADC, others report equivalent survival outcomes between these subtypes [69,70,71]. Overall, while miR-145-5p downregulation may not have directly influenced VTE development in our cohort, it appears to act as a surrogate marker of tumor characteristics and biological behavior, emphasizing its complex and context-dependent role in CC.

Concerning miR-23a-3p and miR-125b-5p, their expression levels were significantly associated with patient characteristics. Low-to-medium miR-23a-3p expression was more frequently observed in patients with early-stage CC (ꭓ^2^ test, *p* = 0.025). While the relationship between circulating miR-23a-3p and CC stage is not fully established, prior studies suggest that miR-23a promotes CC progression by suppressing *TFPI2*, a factor whose inhibition is associated with increased tumor aggressiveness and more advanced disease of CC. Furthermore, high miR-23a levels have also been reported in metastatic fibroblasts [52]. Likewise, elevated miR-23a expression in gastric cancer correlates with advanced stage, lymph node metastasis and higher invasion [72], supporting its pro-tumorigenic role.

For miR-125b-5p, lower expression levels were more common in patients with CSCC (ꭓ^2^ test, *p* = 0.018). This aligns with previous findings showing miR-125b-5p downregulation in HPV16-positive CSCC and high-grade squamous intraepithelial lesions compared to normal cervix tissue [73]. Similarly, in ESCC, reduced miR-125b-5p levels have been linked to HPV infection, and transfection with HPV16E6 is reported to decrease miR-125b-5p expression in ESCC cells [74]. Further investigation is needed to clarify the biological significance of these parallels between CSCC and ESCC.

Lastly, low plasmatic levels of miR-616-3p, along with low-to-medium levels of miR-23a-3p, showed a positive effect on patient OS in the ADC subgroup (log-rank test, *p* = 0.039 for both). This may be attributed to the specific biology of ADC, which typically exhibits higher *vascular endothelial growth factor* (*VEGF*) expression compared to CSCC, making VEGF a more relevant factor in this subtype [75]. VEGF promotes endothelial cell proliferation and angiogenesis but also induces *TFPI2* expression as a negative feedback mechanism. TFPI2 inhibits endothelial cell migration and, at higher concentrations, can suppress VEGFR-2 activity and mitogen-activated protein kinase (MEK)/extracellular signal-regulated kinase (ERK) signaling, exerting anti-angiogenic effects [51,76]. *VEGF-A* overexpression in ADC has been linked to tumor aggressiveness and poorer prognosis [77]. In this study, the downregulation of miR-23a-3p and miR-616-3p in ADC patients was correlated with a less aggressive tumor phenotype. A possible explanation is that, since both miRNAs target *TFPI2*, their reduced levels could lead to an increase in TFPI2, expression which could counteract VEGF-driven angiogenesis. Consequently, higher TFPI2 activity could suppress angiogenesis, and, in this context, improve patient survival.

Within ADC cases, a co-expression pattern was observed for miR-23a-3p, miR-145-5p and miR-616-3p, with patients showing consistent expression levels across all three miRNAs. Given their similar associations with patient OS, their shared biological roles were explored in an in silico analysis. Pairwise comparisons of predicted targets showed that miR-23a-3p and miR-145-5p shared the most targets (N = 10), whereas miR-23a-3p and miR-616-3p, as well as miR-145-5p and miR-616-3p, each shared three targets. Functional enrichment analyses revealed that these shared targets were significantly associated with molecular functions, cellular components and biological processes relevant to tumor biology. DISEASE and TISSUE enrichment analyses further linked these targets to cervical carcinoma, the female reproductive system and vascular diseases. Additionally, Reactome and KEGG pathway analyses highlighted their involvement in oncogenic and pro-thrombotic signaling pathways, including transcriptional dysregulation in cancer, viral carcinogenesis and microRNAs in cancer. Collectively, these results suggest that the shared targets of miR-23a-3p, miR-145-5p and miR-616-3p converge on regulatory roles that integrate cervical tumorigenesis and potentially CAT. However, to confirm and complement the in silico analysis, in vitro and in vivo studies are mandatory, particularly considering the scarce evidence linking these miRNAs to CAT pathogenesis.

This study has some limitations that need to be acknowledged. First, as a retrospective study, it was constrained in its ability to collect relevant data, including comprehensive information on other potential risk factors such as oral contraceptive use, pregnancy and comorbidities. Additionally, VTE was not actively screened, raising the possibility of underdiagnosis, particularly for asymptomatic events. The absence of a healthy control group further limits the capacity to contextualize the observed associations against baseline physiological conditions. Also, this prevents the evaluation of whether the ncRNA dynamics studied are specific to CC-related CAT pathogenesis. Another limitation was the relatively small sample size, most likely making the study statistically underpowered, particularly when focusing on the number of VTE patients (N = 9). This is due to the rarity of both CC and CC-related VTE cases at our institution. These limitations emphasize the need for larger, prospective studies with standardized data collection and appropriate control groups to better elucidate the relationship between CC and venous thrombogenesis.

Despite the constraints, the study also presents interesting strengths. Namely, the cohort was relatively homogeneous, enhancing the internal validity of the findings and increasing confidence in the observed associations. Moreover, this study provides a novel contribution by focusing on circulating miRNAs in blood samples, rather than limiting analysis to CC tissues, as is common in most studies. By assessing miRNA profiles in circulation, this research highlights the potential of these highly stable molecules to be utilized in minimally invasive liquid biopsy approaches in CC patients, offering promising opportunities for disease risk assessment and prognostication.

## 4. Materials and Methods

### 4.1. Population Description

A retrospective cohort study was conducted involving adult patients from the North region of Portugal, all of whom had a confirmed diagnosis of CC. These individuals were admitted to the Clinic of Gynecology of the Portuguese Institute of Oncology of Porto (IPO Porto) between May 2017 and October 2021 to receive first-line treatment. Inclusion criteria comprised patients with CC classified between stages IB2 and IVA and those who were treated with CCRT as the primary treatment approach. The CCRT protocol consisted of weekly doses of cisplatin at 40 mg/m^2^ administered during external radiotherapy with or without subsequent brachytherapy. Patients were excluded if they met any of these conditions: (1) younger than 18 years old, (2) had undergone surgery, considered a risk factor for CAT, at least six months prior to CCRT initiation, or (3) were only seeking a second medical opinion. In total, 69 patients were involved in this study, with a mean follow-up duration of 47.18 ± 2.11 months. All participants provided written informed consent aligned with the principles of the Helsinki Declaration. This study was approved by the ethics committee at IPO Porto (CES IPO:287A/014).

Demographic data, clinicopathological characteristics, and follow-up information were obtained from the patient’s medical records. Tumor staging was determined using the FIGO system [78]. CAT status was also established based on medical records. Notably, no active screening was conducted. A description of the study cohort characteristics is given in Table 3.

### 4.2. Sample Collection and Processing

Before the initiation of CCRT protocol, peripheral blood samples of each patient were collected using a standard venipuncture method. Samples were obtained in ethylenediaminetetraacetic acid (EDTA)-coated vacuum tubes and centrifuged at 3000 rpm for 5 min at room temperature to isolate the plasma fraction. The plasma samples were then stored at −80 °C until the moment of RNA extraction.

### 4.3. MiRNA Selection

MiRNAs were selected based on an extensive literature review aimed at identifying the most relevant candidates implicated in VTE, both in the general population and among cancer patients [29]. In parallel, an exhaustive review of the existing literature on miRNAs involved in cervical carcinogenesis was conducted. MiRNAs were included based on the following criteria: (1) regulatory role in hemostasis; (2) documented involvement in VTE; (3) detection in blood circulation; and (4) established association with cervical carcinogenesis. Based on these inclusion parameters, hsa-miR-20a-5p, hsa-miR-23a-3p, hsa-miR-125b-5p, hsa-miR-145-5p, and hsa-miR-616-3p were selected.

MiR-20a-5p has been shown to regulate both *F3* (gene encoding TF) [57] and *TFPI2*, thereby modulating hemostasis and thrombosis [56]. In CC, it is frequently upregulated in both tissue and serum samples [79,80], where it promotes tumor growth, invasion and autophagy, through the targeting of *Runt-related transcription factor 1* (*RUNX1*) [81], *Autophagy-related protein 7* (*ATG7*), *Tissue Inhibitor of Metalloproteinase 2* (*TIMP2*) [65], *Thrombospondin 2* (*THBS2*) [82], and *Programmed cell death 6* (*PDCD6*) [83]. Similarly, miR-23a-3p and miR-616-3p also target *TFPI2*, influencing fibrinolysis and thrombus stability [31,49,50,56,84]. The former generally acts as an oncogene in several cancers, particularly in pancreatic carcinoma [49]. However, it is downregulated in CC, resulting in *TFPI2* overexpression [85]. As for miR-616-3p, it has been linked to increased CAT susceptibility in ovarian cancer patients [56]. Yet, its role in CC has not been reported.

MiR-125b-5p has a recognized role in thrombotic disease, where its elevated circulating levels positively correlate with platelet count and ischemic stroke severity suggesting a pro-thrombotic function [86,87]; however, in cancer, miR-125b exhibits context-dependent roles, acting as both an oncogene and a tumor suppressor [88]. In CC, this miRNA is downregulated by HPV infection, a mechanism that facilitates viral persistence and carcinogenesis [61,89,90].

Another key regulator of the *F3* is miR-145 [48]. In the general population (cancer-free individuals), reduced levels of this miRNA have been observed in VTE patients, likely reflecting activation of the coagulation cascade and thrombus formation [48,91]. Beyond TF, miR-145 has also been reported to target other hemostasis-related genes, including *F11* (gene encoding *FXI*) [92,93] and *SERPINE1* (gene encoding *PAI-1*) [94], where elevated plasma levels of these proteins’ expression have been associated with a higher risk of VTE occurrence [92,94,95]. In CC, miR-145 downregulation in both tissues and plasma correlates with poor prognosis and aggressive clinical features [55]. Functionally, miR-145 suppresses cervical carcinogenesis through targeting *SMAD-Interacting Protein 1* (*SIP1*) [96], *Helicase-Like Transcription Factor* (*HLTF*) [97], *Wnt Family Member 2B* (*WNT2B*) [98], *Regulators of Calcineurin 3* (*RCAN3*) [99] and *Fascin actin-bundling protein 1* (*FSCN1*) [100]. Taken together, these five miRNAs converge on the regulation of coagulation and tumor progression, positioning them as strong candidates for the purpose of the study.

### 4.4. Total RNA Extraction

Before RNA extraction, previously stored plasma samples were thawed on ice and centrifuged for 4 min at 2000 rpm to remove residual cellular debris and platelets, as previously described [56]. Moving forward, total RNA was extracted from the samples using the MagMAX™ mirVana™ Total RNA Isolation Kit (CAT A27828, Thermo Fisher Scientific, Waltham, MA, USA), according to the manufacturer’s instructions. The extraction procedure was carried out using a KingFisher™ Duo Prime Magnetic Particle Processor (Thermo Fisher Scientific, Waltham, MA, USA). The concentration and purity of the extracted plasma RNA were assessed using a NanoDrop Lite spectrophotometer (Thermo Fisher Scientific, Waltham, MA, USA). The RNA samples presented variable concentrations (8–25 ng/µL). Before cDNA synthesis, the samples were adjusted to a final concentration of 8 ng/µL. Regarding the RNA purity (A260/280 ratio), it varied between 1.20 and 1.35, which is expected when working with human plasma. After quantification, RNA samples were kept at −80 °C.

### 4.5. cDNA Synthesis

Complementary DNA (cDNA) was synthesized from 40 ng of RNA *per* sample using the TaqMan™ MicroRNA Reverse Transcription Kit (CAT 4366596, Thermo Fisher Scientific, Carlsbad, CA, USA), following the manufacturer’s instructions. Sequence-specific stem-loop reverse transcription primers were used for the following targets: hsa-miR-20a-5p (assay ID 000580), hsa-miR-23a-3p (assay ID 000399), hsa-miR-125b-5p (assay ID 000449), hsa-miR-145-5p (assay ID 002278), hsa-miR-451a (assay ID 001141), hsa-miR-616-3p (assay ID 002414), hsa-miR-1228-3p (assay ID 002919) and U6 snRNA (assay ID 001973). Each 10 μL reaction contained 5 μL of RNA and 5 μL of reaction mix. The latter included 10×RT Buffer, dNTP Mix, RT, RNAse inhibitor and the stem-loop RT primers. Reverse transcription was performed in Mycycler™ Thermal Cycler (Bio-Rad Laboratories, Hercules, CA, USA) under the following cycle conditions: 30 min at 16 °C, 60 min at 42 °C and 10 min at 85 °C [56]. Negative controls (lacking RNA) were included in all reverse transcription reactions to assess potential contamination.

### 4.6. Relative Quantification of miRNAs

Quantification of miRNA expression was performed by real-time quantitative polymerase chain reaction (RT-qPCR) using a StepOnePlus™ qPCR system (Applied Biosystems^®^, Foster City, CA, USA). Each PCR reaction was carried out in a final volume of 10 μL, comprising 5.0 μL of 2× TaqMan™ Fast Advanced Master Mix (Applied Biosystems^®^, Foster City, CA, USA), 2.5 μL of nuclease-free water, 0.5 μL of 20× specific TaqMan™ MicroRNA Assays for hsa-miR-20a-5p (assay ID 000580), hsa-miR-23a-3p (assay ID 000399), hsa-miR-125b-5p (assay ID 000449), hsa-miR-145-5p (assay ID 002278), hsa-miR-451a (assay ID 001141), hsa-miR-616-3p (assay ID 002414), hsa-miR-1228-3p (assay ID 002919) and U6 snRNA (assay ID 001973), along with 2.0 μL of cDNA template.

According to previous studies measuring miRNA expressions in human plasma samples, U6 snRNA and miR-1228-3p were tested as housekeeping genes [56,101]. Beyond its potential link to CAT and CC prognosis, miR-23a-3p—known for its stability in plasma samples upon hemolysis—and miR-451a—abundantly expressed in red blood cells—were employed as indicators to evaluate the presence of hemolysis [102]. A ΔCq value (hsa-miR-23a-3p–hsa-miR-451a) greater than 8 indicates a high likelihood of hemolysis [56,102,103]. Since three samples exceeded this threshold, only 66 were included in the miRNA expression analyses.

PCR amplification was performed under the following thermal cycling conditions: 2 min at 50 °C, 10 min at 95 °C, followed by 45 cycles of 15 s at 95 °C and 1 min at 60 °C. Target and reference gene expression levels were quantified for each sample on the same reaction plate. All quantifications were performed in triplicate, and negative controls (lacking cDNA) were included in every reaction for quality assurance. Cycle threshold (Ct) values with a standard deviation (SD) higher than 0.5 were not included in the analysis. The Thermo Fisher Connect platform (Thermo Fisher Scientific, Waltham, MA, USA) was used to set consistent baseline and threshold values across all plates.

### 4.7. Statistical Analysis

Data were analyzed using IBM SPSS Statistics for Windows (version 29, IBM Corp., Armonk, NY, USA), while graphical representations were generated with GraphPad Prism (version 9.0.0, GraphPad Software Inc., La Jolla, CA, USA). The Livak method was employed to determine the miRNA’s normalized relative expression. The miR-1228-3p was deemed the most suitable endogenous control, given its stable expression, which is consistent with a previous study of our research group using plasma samples [56].

Given the lack of previous studies exploring the role of the evaluated miRNAs in the context of CC-related CAT, the expression of each miRNA was analyzed considering four categorized expression profiles: (1) samples were divided into low and high expression groups using the median expression value as a threshold; (2) samples separated into three categories—low, intermediate, and high expression—based on terciles; (3) the first and second terciles were grouped as low expression and the third tercile was classified as high expression; and (4) the first tercile was deemed the low expression group, while the second and third terciles were combined as high expression. All these profiles were considered in analyses where miRNA expression was treated as a categorical variable. The same grouping method was previously applied in other studies of our research group [56,104].

Data normality was assessed using either the Shapiro–Wilk test or the Kolmogorov–Smirnov test, depending on the sample size (N ≤ 50 and N > 50, respectively). Based on the outcome of the normality test, correlations between miRNA expression levels were examined using either Pearson’s correlation coefficient (for normally distributed data) or Spearman’s rank correlation coefficient (for non-parametric data). Correlations were deemed noteworthy when the *p*-value was less than 0.05 and the correlation coefficient was ≥0.500. Correlation strength was interpreted using the following categories: coefficients between 0.500 and 0.700 were considered moderate, 0.700–0.900 strong, and ≥0.900 very strong [40,41,42].

The relationship between miRNA expression and patients’ demographic or clinical-pathological features (categorical variables) was evaluated by applying the chi-square test (χ^2^ test). Age categories (<52 vs. ≥52 years) were based on the mean age upon CC diagnosis, given that the variable followed a normal distribution (Kolmogorov–Smirnov test, *p* > 0.05). Differences in miRNA expression according to VTE occurrence [no vs. yes] were assessed using either the Mann–Whitney U test or an unpaired *t*-test, depending on data normality. These comparisons were confirmed using the χ^2^ test and logistic regression analysis.

The impact of miRNA expression on OS was assessed in the entire cohort and considering patients’ age (<52 vs. ≥52 years), histological type (CSCC vs. ADC vs. others), FIGO stage (<IIB vs. ≥IIB) and VTE status (no vs. yes). OS was defined as the period between CC diagnosis and patient death due to all causes or the last follow-up date. Kaplan–Meier survival analysis was used to generate survival curves, while differences between groups were evaluated using the log-rank test or Tarone-Ware test, depending on the proportionality and timing of events across groups. All tests were two-tailed with a 5% significance threshold.

### 4.8. In Silico Analysis

The biological relevance of the relevant miRNAs was investigated through a comprehensive in silico analysis. All validated miRNA targets were obtained using the miRTargetLink 2.0 database (https://ccb-compute.cs.uni-saarland.de/mirtargetlink2/; last accessed on 9 August 2025). PPI networks were then constructed via the STRINGapp Protein Query from Cytoscape 3.10.3, focusing on physical interactions. Functional enrichment analysis was subsequently performed, considering a false discovery rate (FDR) threshold of <0.01 and filtering out redundant terms with a similarity cut-off of 0.5. The top 20 enriched terms, if available, were retrieved for each category, including GO terms, disease (pathological processes), tissues (tissue-specific expression), KEGG pathways, and Reactome pathways.

## 5. Conclusions

CC patients are commonly affected by CAT, fueling tumor progression and worsening prognosis. Thus, identifying CC biomarkers to aid in decision-making is crucial [105,106]. MiRNAs are promising candidates due to their stability in liquid biopsies and their established roles in the regulation of both thrombosis-related pathways and CC biology [107,108]. MiRNA profiling could thus offer new opportunities for disease monitoring and therapeutic strategies in CC patients at increased thrombotic risk. Hence, this study evaluated the plasma expression levels of miRNAs involved in venous thrombogenesis and cervical carcinogenesis in a cohort of CC patients. According to the findings, VTE occurrence negatively affects the prognosis of CC patients. Among the miRNAs analyzed, miR-20a-5p and miR-145-5p demonstrated the most evident links with the studied diseases and clinical features. The former was the only miRNA significantly associated with CAT occurrence, suggesting its potential role as a predictive biomarker of thrombotic risk in CC. This could be useful to aid in thromboprophylaxis. Interestingly, besides its role in CAT, miR-20a-5p upregulation was linked to ADC histology, indicating a context-dependent function in cervical tumorigenesis. Regarding miR-145-5p, it exhibited a prognostic value in ADC patients and those with CAT events, also reflecting its context-specific role. Finally, in ADC patients, miR-23a-3p, miR-145-5p and miR-616-3p were found to have a similar influence on OS, highlighting their potential utility as prognostic biomarkers within this histological context. Overall, these preliminary findings emphasize the relevance of miRNA profiling as tools to better understand CC pathogenesis, assess thrombotic risk and improve prognostication. Moving forward, to complement the in silico analysis, future studies should aim to investigate the molecular mechanisms in CC and CAT involving these miRNAs through in vitro and in vivo studies, allowing the exploration of potential therapeutic targets. In conclusion, the evaluated miRNAs demonstrate functional significance and a predictive biomarker potential in CC, which could enable a more personalized risk assessment and targeted interventions.

## Figures and Tables

**Table 1:** Correlation analysis between miRNA expression levels.

miRNA		miR-20a-5p	miR-23a-3p	miR-125b-5p	miR-145-5p	miR-616-3p
**miR-20a-5p**	Coefficient test	-	0.659 *	0.598	0.662 *	0.555
	*p*-value		<0.001	<0.001	<0.001	<0.001
**miR-23a-3p**	Coefficient test	0.659 *	-	0.546	0.700 *	0.305
	*p*-value	<0.001		<0.001	<0.001	<0.001
**miR-125b-5p**	Coefficient test	0.598	0.546	-	0.660	0.332
	*p*-value	<0.001	<0.001		<0.001	<0.001
**miR-145-5p**	Coefficient test	0.662 *	0.700 *	0.660	-	0.434
	*p*-value	<0.001	<0.001	<0.001		<0.001
**miR-616-3p**	Coefficient test	0.555	0.305	0.332	0.434	-
	*p*-value	<0.001	<0.001	<0.001	<0.001	

Spearman’s rank correlation coefficient test was used when at least one of the miRNAs did not show normal distribution. * Pearson’s rank correlation coefficient test was calculated when both miRNAs followed a normal distribution. According to the literature, the strength of the correlations (Spearman’s ρ and Pearson’s *r* ≥ 0.500 and *p* < 0.05) was classified as follows: moderate (Spearman’s ρ and Pearson’s *r* < 0.700), strong (0.700 ≤ Spearman’s ρ and Pearson’s *r* < 0.900), and very strong (Spearman’s ρ and Pearson’s *r* ≥ 0.900) [40,41,42]. Abbreviation: miRNA, microRNA.

**Table 2:** Pattern of miRNA expression according to patients’ characteristics.

miRNA	Age (Years)		Histology			FIGO Stage	
	<52	≥52	CSCC	ADC	Other	<IIB	≥IIB
	N (%)	N (%)	N (%)	N (%)	N (%)	N (%)	N (%)
**miR-20a-5p**							
**Low**	7 (21.2%)	15 (48.4%)	31 (60.8%)	2 (20%)	0 (0%)	6 (66.7%)	27 (49.1%)
**High**	26 (78.8%)	16 (51.6%)	20 (39.2%)	8 (80%)	3 (100%)	3 (33.3%)	28 (50.9%)
*p*-value	0.043 *		**0.003**			0.332	
**miR-23a-3p**							
**Low**	13 (39.4%)	20 (58.8%)	28 (53.8%)	4 (33.3%)	1 (33.3%)	9 (100%)	36 (62.1%)
**High**	20 (60.6%)	14 (41.2%)	24 (46.2%)	8 (66.7%)	2 (66.7%)	0 (0%)	22 (37.9%)
*p*-value	0.178		0.194			0.025 **	
**miR-125b-5p**							
**Low**	12 (36.4%)	21 (61.8%)	21 (40.4%)	1 (8.3%)	0 (0%)	5 (55.6%)	28 (48.3%)
**High**	21 (63.6%)	13 (38.2%)	31 (59.6%)	11 (91.7%)	3 (100%)	4 (44.4%)	30 (51.7%)
*p*-value	0.067		0.018 *			0.687	
**miR-145-5p**							
**Low**	13 (39.4%)	20 (58.8%)	29 (55.8%)	4 (33.3%)	0 (0%)	8 (88.9%)	25 (43.1%)
**High**	20 (60.6%)	14 (41.2%)	23 (44.2%)	8 (66.7%)	3 (100%)	1 (11.1%)	33 (56.9%)
*p*-value	0.178		**0.027**			**0.011**	
**miR-616-3p**							
**Low**	14 (43.8%)	17 (54.8%)	27 (54%)	4 (40%)	0 (0%)	5 (62.5%)	26 (47.3%)
**High**	18 (56.2%)	14 (45.2%)	23 (46%)	6 (60%)	3 (100%)	3 (37.5%)	29 (52.7%)
*p*-value	0.530		0.070			0.425	

All *p*-values correspond to comparisons between low and high miRNA expression levels (expression profile 1. See Section 4.7. Statistical Analysis). *p*-values marked with * refer to comparisons between low vs. medium–high expression levels, while those marked with ** refer to comparisons between low–medium vs. high expression levels. Abbreviations: miRNAs, microRNAs; CSCC, Cervical Squamous Cell Carcinoma; ADC, Cervical Adenocarcinoma; FIGO, International Federation of Gynecology and Obstetrics.

**Table 3:** Demographic and clinicopathological characterization of CC patients (N = 69).

Variable	N (%)
**Age at CC diagnosis (years)**	51.9 ± 13.4 *
<52	33 (47.8)
≥52	34 (49.3)
Missing data	2 (2.9)
**Histology**	
Squamous Cell Carcinoma	52 (75.4)
Adenocarcinoma	12 (17.4)
Other	3 (4.3)
Missing data	2 (2.9)
**FIGO Stage**	
<IIB	9 (13.0)
≥IIB	58 (84.1)
Missing data	2 (2.9)
**VTE Status**	
No	58 (84.1)
Yes	9 (13.0)
Missing data	2 (2.9)

* Mean ± standard deviation. Abbreviations: CC, Cervical Cancer; FIGO, International Federation of Gynecology and Obstetrics; N, number; VTE, Venous Thromboembolism.

## Data Availability

The data presented in this study are available upon request from the corresponding author.

## Abbreviations

The following abbreviations are used in this manuscript:

ADC	Cervical Adenocarcinoma
ATG7	Autophagy-related protein 7
CAT	Cancer-Associated Thrombosis
CC	Cervical Cancer
CCRT	Concurrent chemoradiotherapy
cDNA	Complementary DNA
CI	Confidence Interval
CSCC	Cervical Squamous Cell Carcinoma
Ct	Cycle threshold
EAC	Esophageal Adenocarcinoma
EC	Esophageal Cancer
EDTA	Ethylenediaminetetraacetic acid
ERK	Extracellular signal-regulated kinase
ESCC	Esophageal Squamous Cell Carcinoma
F3	Coagulation Factor 3
FDR	False Discovery Rate
FIGO	International Federation of Gynecology and Obstetrics
FSCN1	Fascin actin-bundlin protein 1
FVIIIa	Activated Coagulation Factor VIII
FXa	Activated Coagulation Factor X
FXI	Coagulation Factor XI
FXIa	Activated Coagulation Factor XI
GO	Gene Ontology
HLTF	Helicase-Like Transcription Factor
HPV	Human Papillomavirus
HR	Hazard Ratio
IPO Porto	Portuguese Institute of Oncology of Porto
MEK	Mitogen-activated protein kinase
mRNAs	Messenger RNAs
miRs or miRNAs	microRNAs
N	number
ncRNAs	Non-coding RNAs
ns	Non-significant
OS	Overall Survival
PAI-1	Plasminogen Activator Inhibitor-1
PDCD6	Programmed Cell Death 6
pKLK	Plasma Kallikrein
PPI	Protein–protein interaction
RCAN3	Regulators of Calcineurin 3
RT-qPCR	Real-Time quantitative Polymerase Chain Reaction
RUNX1	Runt-related Transcription Factor
SD	Standard Deviation
SIP1	SMAD-Interacting Protein 1
TF	Tissue Factor
TFPI1	Tissue Factor Pathway Inhibitor 1
TFPI2	Tissue Factor Pathway Inhibitor 2
THBS2	Thrombospondin 2
TIMP2	Tissue Inhibitor of Metalloproteinase 2
VEGF	Vascular Endothelial Growth Factor
VTE	Venous Thromboembolism
WNT2B	Wnt family member 2B

## Appendix A

**Table A1:** Validated targets of miR-23a-3p, miR-145-5p and miR-616-3p according to MirTargetLink 2.0.

Validated Targets According to MirTargetLink 2.0 *						
miR-23a-3p			miR-145-5p			miR-616-3p
*APAF1*	*DNAJC21*	*PTPDC1*	*ABCC1*	*NAIP*	*IVNS1ABP*	*PON1*
*ATAT1*	*DOCK4*	*PTPN14*	*ABHD17C*	*NANOG*	*KIF21A*	*TFPI2*
*CDH1*	*DSTN*	*QSER1*	*ABRACL*	*NDRG2*	*LMNB2*	*BBOX1*
*CHUK*	*DYNC2LI1*	*RNF111*	*ADAM17*	*NDUFA4*	*MAP1LC3B*	*C5orf46*
*CXCL12*	*DYNLL2*	*RNF38*	*ADD3*	*NEDD9*	*MAP2K4*	*CAPRIN2*
*CXCL8*	*EGLN3*	*RNMT*	*AKR1B10*	*NFATC1*	*MAP3K11*	*CARNS1*
*FANCG*	*EN2*	*RPL7L1*	*ALDH3A1*	*NIPSNAP1*	*MAP3K3*	*CCSAP*
*FAS*	*ERBIN*	*RPP14*	*ALPPL2*	*NRAS*	*MAP4K2*	*CDK4*
*FOXA1*	*ETNK1*	*RPRD2*	*ANGPT2*	*NUDT1*	*MUC19*	*CEP350*
*FOXO3*	*FAM222B*	*RPS4X*	*AP1G1*	*PAK4*	*MUC4*	*CNNM2*
*FZD5*	*FAM91A1*	*RPS5*	*APH1A*	*PARP8*	*NDUFS2*	*COIL*
*G6PC*	*FASTKD1*	*RPSAP58*	*ARF6*	*PIGF*	*NR1D2*	*CREBZF*
*GJA1*	*FASTKD5*	*S100A7A*	*ARL6IP5*	*PODXL*	*NUFIP2*	*CSGALNACT1*
*GLS*	*FAU*	*SCAMP2*	*BNIP3*	*POU5F1*	*NUP43*	*CXCL5*
*HES1*	*FBN2*	*SDHD*	*BRAF*	*PPP3CA*	*ORC4*	*DCTPP1*
*HMGB2*	*FKBP4*	*SEMA6D*	*C11orf65*	*PTP4A2*	*P4HA1*	*DLGAP3*
*HMGN2*	*FLNA*	*SEPT2*	*CAMK1D*	*PXN*	*PADI1*	*FSHB*
*HNF1B*	*FNIP1*	*SERINC3*	*CBFB*	*ROBO2*	*PANK1*	*HLA-DOB*
*HOXB4*	*FOXM1*	*SESN2*	*CCDC43*	*ROCK1*	*PDGFD*	*IGFBP5*
*HSP90AA1*	*FOXR2*	*SLC27A4*	*CD28*	*RPA1*	*PHACTR2*	*INTS6*
*IL6R*	*FSD1L*	*SLC35G3*	*CD40*	*RPS6KB1*	*PLAGL2*	*KMT2A*
*IRF1*	*FUT4*	*SLC6A15*	*CD44*	*RREB1*	*PLEKHM1*	*LGALS3BP*
*KLF3*	*GGA3*	*SLC6A6*	*CDH2*	*RTKN*	*PNMA3*	*LPAR2*
*LAMP1*	*GMPS*	*SNTG1*	*CDK4*	*SENP1*	*PRDM2*	*MAL2*
*LDHA*	*GNAI3*	*SOCS6*	*CDK6*	*SERINC5*	*PSAT1*	*MC2R*
*LDHB*	*GNB2*	*SOD2*	*CDKN1A*	*SERPINE1*	*RAB3IP*	*MED17*
*LPAR1*	*GRK2*	*SPTY2D1*	*CEP19*	*SET*	*RBM18*	*MIEF1*
*LRP5*	*GRTP1*	*SSR1*	*CFTR*	*SMAD2*	*REL*	*MIER3*
*MEF2C*	*GTF3C4*	*SSRP1*	*CLINT1*	*SMAD3*	*RPS6KA3*	*MRO*
*MT2A*	*HAPLN1*	*STAMBPL1*	*COL5A1*	*SOCS7*	*RRAGC*	*MTHFD1*
*MYH1*	*HCFC1*	*STARD7*	*CRNDE*	*SOX2*	*SAMD5*	*PAFAH1B1*
*MYH2*	*HIST1H3B*	*STS*	*CTGF*	*SOX9*	*SESN2*	*PRKAG1*
*MYH4*	*HIVEP1*	*STT3B*	*CTNND1*	*SP1*	*SLC16A10*	*PRPF4B*
*NEK6*	*HMGCS1*	*SWAP70*	*DDC*	*SP7*	*SLC16A5*	*PSMA2*
*POU4F2*	*HNRNPUL1*	*TBL2*	*DDX17*	*SPTBN1*	*SLC22A9*	*PTMA*
*PPARGC1A*	*HRG*	*TCF25*	*DDX6*	*SPTLC1*	*SLC26A2*	*QSER1*
*PPP2R5E*	*HS2ST1*	*THUMPD3*	*DFFA*	*SRGAP1*	*SMAD4*	*RBM26*
*PTEN*	*IMPDH2*	*TLR6*	*DTD1*	*STAT1*	*SMAD5*	*RBM43*
*PTPN11*	*ITPKC*	*TMED7*	*E2F3*	*SWAP70*	*SMIM17*	*SERTAD2*
*RGS5*	*JMJD1C*	*TMEM170A*	*EGFR*	*TGFB2*	*SNTB1*	*SFMBT2*
*SMAD3*	*KDM3B*	*TNFAIP3*	*EIF4E*	*TGFBR2*	*SNX24*	*SIK1*
*SMAD5*	*KIAA1210*	*TNFAIP8*	*EPAS1*	*TIRAP*	*SOX11*	*SLFN12*
*SPRY2*	*KIAA1551*	*TNPO1*	*ERG*	*TMEM9B*	*SRPX2*	*SLFN12L*
*ST7L*	*KIF20A*	*TNRC6A*	*ETS1*	*TNFSF13*	*TGFBI*	*SMAD4*
*STAT3*	*KIF22*	*TOPBP1*	*F11R*	*TPM3*	*THSD7A*	*TMEM59*
*TERF2*	*KLF10*	*TOR1AIP1*	*FAM3C*	*TPRG1*	*TMOD3*	*TOR4A*
*TMEM64*	*KLF12*	*TPM3*	*FAM45A*	*TSPAN6*	*TNR*	*TRMT112*
*TOP1*	*LEMD2*	*TRIM59*	*FLI1*	*TUG1*	*UBR7*	*USP37*
*TSC1*	*LMAN2*	*TRIM63*	*FSCN1*	*VEGFA*	*UBXN2A*	*XIAP*
*XIAP*	*LMNB1*	*TRRAP*	*FXN*	*VPS51*	*UNC5D*	*ZNF347*
*ABCD1*	*MAP1B*	*TSNAX*	*GMFB*	*YES1*	*UTP15*	*ZNF35*
*ACSS3*	*MC2R*	*TXNIP*	*GOLM1*	*AAED1*	*VGLL4*	*ZNF460*
*ACTN4*	*MCFD2*	*TXNL4A*	*HDAC11*	*ACTB*	*WASHC2C*	*ZNF850*
*ADAM17*	*MCU*	*UBALD1*	*HDAC2*	*AGPS*	*WSB1*	*PON1*
*ADAM28*	*MGAT5*	*UQCRFS1*	*HLTF*	*AGTRAP*	*ZBTB25*	
*ADAM9*	*MMGT1*	*USP34*	*HMGA2*	*ALG9*	*ZFAND3*	
*ADD3*	*MPP6*	*USP5*	*IFNB1*	*ANKRD28*	*ZFYVE9*	
*ARL6IP1*	*MRPS34*	*VAV3*	*IGF1R*	*AQR*	*ZNF100*	
*ASNS*	*MTAP*	*VCAM1*	*ILK*	*BCLAF3*	*ZNF426*	
*ATP5A1*	*MYC*	*WEE1*	*IRS1*	*BTG1*	*ZNF445*	
*ATXN7L3B*	*MYH10*	*ZBTB10*	*IRS2*	*C1orf27*	*ZNF451*	
*BAZ2A*	*NACC1*	*ZCCHC2*	*ITGB8*	*CCDC80*	*ZNF660*	
*BCAP29*	*NDUFV3*	*ZIK1*	*JADE1*	*CCDC85C*	*ZNF678*	
*BRWD1*	*NLGN4X*	*ZNF117*	*KLF4*	*CLSTN2*	*ZNF772*	
*BSDC1*	*NLK*	*ZNF138*	*KLF5*	*CRAMP1*		
*BTLA*	*NOL11*	*ZNF253*	*KREMEN1*	*CRYBG1*		
*C2orf69*	*NUFIP2*	*ZNF257*	*LYPLA2*	*CSRNP3*		
*C8orf58*	*NUP153*	*ZNF267*	*MAP2K6*	*CTNNBIP1*		
*CAMKV*	*P2RY11*	*ZNF268*	*MCM2*	*CYP2C19*		
*CCL8*	*PAFAH1B3*	*ZNF273*	*MDM2*	*DDI2*		
*CCT5*	*PDCD6IP*	*ZNF275*	*MEST*	*DEK*		
*CD302*	*PDIA6*	*ZNF281*	*MIXL1*	*DMXL1*		
*CELF1*	*PEX26*	*ZNF319*	*MMP1*	*DNAJC28*		
*CENPM*	*PIGM*	*ZNF426*	*MMP12*	*DUSP6*		
*CEP57L1*	*PIK3R1*	*ZNF485*	*MMP14*	*EID2B*		
*CNN2*	*PKM*	*ZNF550*	*MSH3*	*ERBB4*		
*CNOT1*	*PLAG1*	*ZNF578*	*MTDH*	*FZD6*		
*CNOT6*	*PNRC2*	*ZNF669*	*MTMR14*	*HBEGF*		
*CNOT9*	*POM121C*	*ZNF682*	*MUC1*	*HIF1A*		
*CRLS1*	*PPIC*	*ZNF701*	*MYC*	*HIST1H2AH*		
*CSDE1*	*PSAP*	*ZNF704*	*MYO5A*	*HIST1H2BF*		
*DCP2*	*PSMC3*		*MYO6*	*IGFBP5*		
*DDX5*	*PTGFRN*		*MYOCD*	*IMMP2L*		

* Last accessed on 9 August 2025. Colored targets represent the strongly validated targets, meaning confirmed experimentally via Luciferase reporter assay, Western blot, and/or qRT-PCR.
